# A Multidisciplinary Intubation Algorithm for Suspected COVID-19 Patients in the Emergency Department

**DOI:** 10.5811/westjem.2020.5.47835

**Published:** 2020-06-03

**Authors:** Lauren L. Trembley, Adam Z. Tobias, Gwendolyn Schillo, Nicholas von Foerster, Jordan Singer, Samantha L. Pavelka, Paul Phrampus

**Affiliations:** * University of Pittsburgh, Department of Emergency Medicine, Pittsburgh, Pennsylvania; † University of Pittsburgh, Winter Institute for Simulation, Education and Research (WISER), Pittsburgh, Pennsylvania

## Abstract

**Introduction:**

Intubation of patients suspected of having coronavirus disease 2019 (COVID-19) is considered to be a high-risk procedure due to the aerosolization of viral particles. In an effort to minimize the risk of exposure and optimize patient care, we sought to develop, test, provide training, and implement a standardized algorithm for intubating these high-risk patients at our institution.

**Methods:**

We developed an initial intubation algorithm, incorporating strategic use of equipment and incorporating emerging best practices. By combining simulation-based training sessions and rapid-cycle improvement methodology with physicians, nurses, and respiratory therapists, and incorporating their feedback into the development, we were able to optimize the process prior to implementation. Training sessions also enabled the participants to practice the algorithm as a team. Upon completion of each training session, participants were invited to complete a brief online survey about their overall experience.

**Results:**

An algorithm and training system vetted by simulation and actual practice were developed. A training video and dissemination package were made available for other emergency departments to adopt. Survey results were overall positive, with 97.92% of participants feeling confident in their role in the intubation process, and many participants citing the usefulness of the multidisciplinary approach to the training.

**Conclusion:**

A multidisciplinary, team-based approach to the development and training of a standardized intubation algorithm combining simulation and rapid-cycle improvement methodology is a useful, effective process to respond to rapidly evolving clinical information and experiences during a global pandemic.

## INTRODUCTION

The 2019 novel coronavirus first emerged in Wuhan, China, in December 2019 and was declared a pandemic by the World Health Organization (WHO) on March 11, 2020.^1^ As the disease spread rapidly across the globe, healthcare providers who have traditionally been responsible for airway management, including emergency physicians, intensivists, and anesthesiologists, had to quickly adjust routine practices to account for concerns of exposure to, and spreading of, the virus as it was postulated to have stability in aerosolized form.^2^

Airway management with endotracheal intubation is a high-risk and time-sensitive medical task. It is standard practice in emergency medicine training programs to teach a systematic approach to airway management, often enlisting the use of checklists or algorithms.^3^ The multimodal training focuses on motor skills, assessment skills, and decision-making. However, it is uncommon to introduce education simultaneously with a systematic evaluation of iterative process changes associated with what is normally considered routine airway management care. Evaluating necessary process changes that included the complexity involved with standardizing airway, communications, and team-based skills in order to minimize aerosolization of highly infectious viral particles during intubation proved challenging.

Early data from China estimate that 3.2% of confirmed coronavirus disease 2019 (COVID-19) cases developed severe disease requiring endotracheal intubation and positive pressure ventilation at some point in their clinical course.^4^ Due to the potential for aerosolization of patient secretions during this invasive procedure, endotracheal intubation is recognized to be a high-risk procedure in terms of potential exposure and transmission to healthcare providers.^5^

As more cases emerged in the United States, process recommendations regarding intubation were made by various groups.^6^–^9^ Major themes of these recommendations include the use of an N95 respirator or powered air-purifying respirator (PAPR) as part of personal protective equipment (PPE) by all members of the healthcare team with direct patient contact during the procedure. Environmental considerations include the recommended utilization of a negative pressure isolation room for the procedure when possible, as well as minimizing risk by having the fewest number of providers with direct patient contact. Procedural recommendations included having the most experienced provider perform the intubation using video laryngoscopy, rapid sequence induction (RSI), and avoiding the use of non-invasive positive pressure ventilation and bag-valve-mask ventilation (BVM).

While these recommendations provide general guidance and strategies for intubating patients with either confirmed or suspected COVID-19, there is still a need to incorporate these changes at the local level. The risk of aerosolization of viral particles during the procedure requires adaptations to standard airway management algorithms and procedures, based on resources available. Without experience and training with these new methods, and without an established protocol for their implementation, there is potential for suboptimal patient care and increased risk of exposure to the healthcare team. Therefore, training healthcare providers on the new changes will help to avoid uncertainty and confusion, reduce risks of healthcare provider infection, and lead to increased first-pass success for the intubation procedure.

To implement such change, there is a need to develop and implement a stepwise process for intubation of high-risk COVID-19 patients that incorporates the newly published recommendations. Changes to existing emergency department (ED) airway management routines require a multidisciplinary approach, attention to detail, and a rapid-cycle improvement process to guide the development of a new algorithm. Each cycle of testing and training needs to inform necessary changes to the developing algorithm based on the successes and identified areas that did not perform optimally.

**Population Health Research Capsule**

What do we already know about this issue?

*The coronavirus disease 2019 (COVID-19) pandemic has forced healthcare providers to make adaptations to the procedure of intubation to minimize exposure risk*.

What was the research question?

*We sought to develop, test, and implement a standardized intubation algorithm for suspected COVID-19 patients*.

What was the major finding of the study?

*A simulation-vetted algorithm and training system were developed and disseminated across our healthcare system*.

How does this improve population health?

*Our standardized approach to intubation minimizes exposure risk, increases the quality of patient care, and can easily be adapted at other institutions*.

Simulation has previously been identified as a successful tool to educate and serve as a useful framework to evaluate system change to clinical processes,^10^,^11^ as well as teamwork and systems-related training in critical care environments.^12^ Simulation has also been described by our institution and others as a useful modality for rapid development of necessary curriculum and process validation during pandemic preparedness.^13^–^15^

The primary goal of this project was to develop and implement a standard process for intubation of all patients with suspicion for COVID-19 for the ED at our institution, employing a multidisciplinary approach using simulation and a rapid-cycle improvement methodology. We designed our revised approach to incorporate the emerging best practices including 1) minimization of exposure risk to aerosolized patient secretions; 2) optimization of the strategic use of equipment; 3) maximization of first-pass intubation success, 4) enhanced teamwork, communications and patient safety principles; and 5) incorporation of quick access to backup, emergency equipment in case of a difficult airway.

Our primary outcome was to conduct training sessions, develop a modified airway algorithm that had been tested for functional use, and create a deployable training package for dissemination across the EDs of our health system.

## METHODS

### Team and Equipment Deployment

Our algorithm development process was developed around a four-member team that included a physician (DR), two registered nurses (RN1 and RN2), and a respiratory therapist (RT). Three members of the team (DR, RN2, and RT) would participate in the actual procedure while RN1 would serve as logistics support outside the zone of potential contamination. A fifth person, a patient care technician (PCT), could assist RN1 as needed if available.

**Figure 1:**
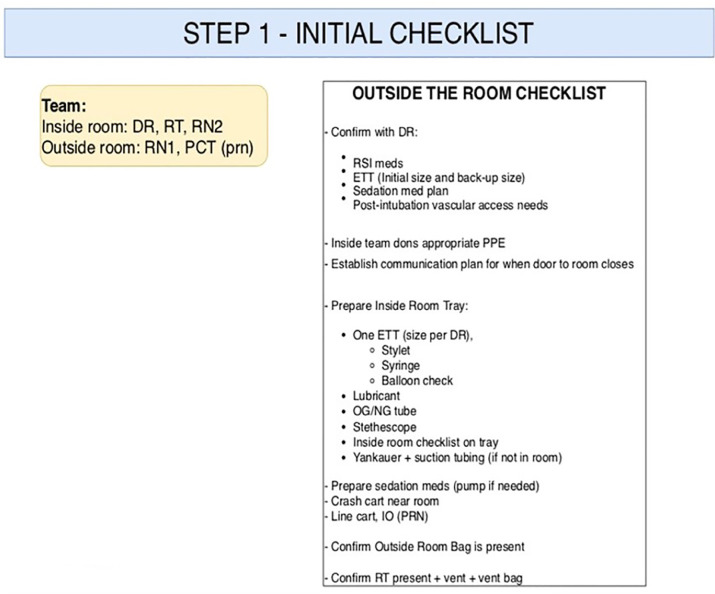
Step 1 of COVID-19 intubation algorithm. *DR*, physician; *RT*, respiratory therapist; *RN*, registered nurse; *PCT*, patient care technician; *RSI*, rapid sequence intubation; *ETT*, endotracheal tube; *PPE*, personal protective equipment; *OG/NG*, orogastric, nasogastric; *IO*, Intraosseous infusion; *PRN*, as necessary.

The first step in development of our procedural algorithm was to compile a list of standard equipment needed for intubations of infected or suspected COVID-19 patients. We first identified the minimum standard equipment and medications that would need to be prepared to enter the procedural area. The equipment is prepared on a standard bedside tray and minimized to prevent confusion and unnecessary contamination or equipment waste. The equipment to be prepared on the tray was organized into a bag labeled “Inside Bag” to indicate the contents were to go into the procedure room. Inside items included standard intubating equipment, listed in Figure 1.

A second bag, designated as the “Outside Bag,” contained items that were to be staged immediately outside the room in which the procedure was to occur and contained what would be historically considered backup equipment for difficult airways. Outside items consisted of a cricothyrotomy kit, I-gel (Intersurgical, Berkshire UK), and gum elastic bougie. The I-gel was selected as the primary rescue device mainly due to its ease of insertion compared to other supraglottic devices. The Outside Bag is designed to remain outside of the room with the belief that it would be uncommonly needed and could remain unopened to avoid unnecessary equipment waste.

A third bag, designated the “Vent Bag,” contains items needed to initially confirm tube placement and would be carried into the treatment area along with the ventilator, and then assembled by the RT. Equipment in this bag included BVM, viral filter, PEEP (positive end expiratory pressure) valve, and colorimetric carbon dioxide (CO2) detector. These bags were attached to each ventilator to ensure easy access and availability.

The bags of equipment were pre-assembled and stored in the designated treatment area in our ED for intubating patients suspicious of COVID-19, ensuring that they were readily available and easy to access.

### Algorithm Development and Team Roles

The initiation of our procedure is triggered when the physician decides that a patient’s clinical condition requires intubation. The core management team for the patient is quickly established and the DR, RT, and RN2 don appropriate PPE. Simultaneously, RN1 begins following a checklist to accomplish STEP 1 in our procedure (Figure 1). STEP 1 focuses on preparing medications for rapid sequence induction (RSI) and post-intubation sedation, verifying that “inside items” are present, preparing the endotracheal tube (ETT) selected by the physician, and anticipating any additional procedures to be completed after intubation, such as central line placement.

**Figure 2:**
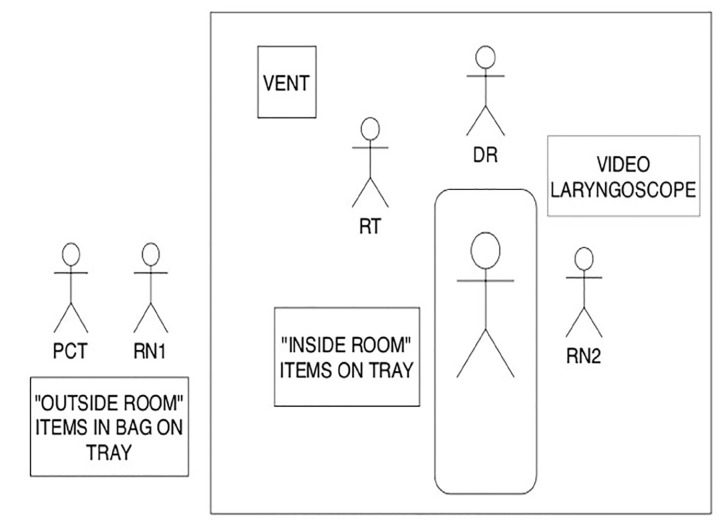
Layout of core management team and equipment for intubation. *RT*, respiratory therapist; *DR*, physician; *RN*, registered nurse; *PCT*, patient care technician.

Once STEP 1 is completed, the DR, RN2, and RT proceed inside the room. The DR is responsible for transporting the video laryngoscope and blades and setting up the equipment. The “inside items” (that had been prepared by RN1) are rolled in by RN2, and the ventilator and Vent Bag are transported in and set up by the RT. Our final idealized placement of equipment and providers is in Figure 2.

**Figure 3:**
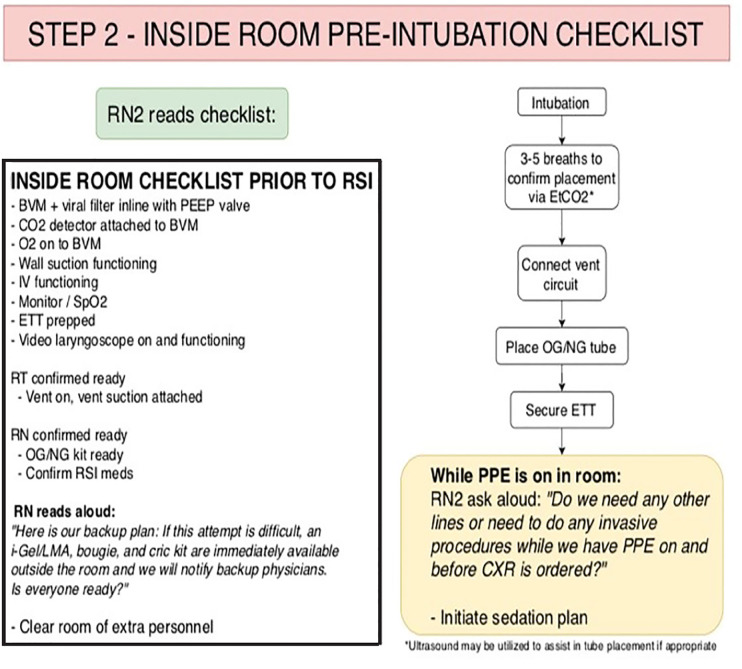
Step 2 of COVID-19 intubation algorithm. *BVM*, bag-valve mask; *PEEP*, positive end expiratory pressure; *CO_2_*, carbon dioxide; *O_2_*, oxygen; *SpO_2_*, peripheral capillary oxygen saturation; *RT*, respiratory therapist; *RN*, registered nurse; *LMA*, laryngeal mask airway; *RSI*, rapid sequence intubation; *ETT*, endotracheal tube; EtCO_2_, end-tidal carbon dioxide; *PPE*, personal protective equipment; *OG/NG*, orogastric/nasogastric; *CXR*, chest radiograph.

The RN2 then reads the pre-intubation checklist, which begins STEP 2 (Figure 3). The checklist serves as a time-out to ensure necessary equipment is present and functioning. After the initial checklist is completed, RN2 then reads the script (Figure 3), which serves as a reminder to the team about the backup plan and equipment that is immediately available, should intubation prove difficult.

The DR then performs the intubation. To minimize aerosolization of secretions, pre-oxygenation is delivered by face mask oxygen at 10–12 liters (L) per minute (min) with an additional 5–6 L/min of oxygen delivered via nasal cannula, which remains in place upon removal of face mask. This method provides apneic oxygenation, reducing the potential need for BVM or positive pressure ventilations. If the intubation is successful the BVM, pre-fitted with a viral filter and a CO_2_ detector, is connected and up to five shallow breaths are given to confirm tube placement with the colorimetric device.

The BVM is then disconnected, and the DR quickly covers the disconnected ETT with his or her thumb while the patient is hooked up to the vent circuit. The RT then holds the ETT while the DR places an orogastric or nasogastric tube. The DR then holds the tube while the RT places the tube-holder and secures the ETT. Once the tube has been secured, RN2 will then read the script (Figure 3), prompting the DR to place any additional lines or other invasive procedures before doffing PPE. This ensures that all lines will be placed prior to radiograph confirmation, attempting to minimize exposure to radiology technicians and conserve PPE. The pre-identified, post-intubation sedation plan will then be implemented.

In the event of a difficult airway or failed first attempt, we incorporated an early activation of a backup plan (that is appropriate for the given facility) into our algorithm. Thus, if the DR requests the Outside Bag, RN1 would also call for additional help. If the airway proved difficult and intubation is not successful within a reasonable time, or if the patient decompensated, we encouraged placement of the I-gel backup device and ventilation through the I-gel until additional resources arrived. This is based upon previous studies demonstrating the I-gel to be the quickest device to be used to secure the airway while wearing PPE.^16^ The DR also has the option to perform a cricothyrotomy if clinically indicated.

### Training, Refinement and Implementation

Upon completion of the initial version of the intubation algorithm, we partnered with the Winter Institute for Simulation, Education and Research (WISER) to conduct simulation-based training sessions. WISER is the simulation institute of the University of Pittsburgh and the UPMC Health System and is accredited by the Society for Simulation in Healthcare in the areas of Teaching/Education, Assessment, Research, and Systems Design. The simulation training sessions were strategically designed to teach a refresher of airway management as modified for the pandemic, but also to study our new processes, incorporating the necessary teamwork and communications to allow for rapid optimization. In addition to standard simulation-based training, we employed the Plan-Do-Study-Act (PDSA) rapid-cycle improvement process to evaluate the need for refinements of our process changes as well as our educational content.

We held seven days of multiple one-hour sessions for multidisciplinary training, deliberate practice, and process refinement. Participation was voluntary. DRs were recruited via email and could select a convenient time over the available training days. RNs and RTs were recruited from those working in the department, as identified by nursing and RT leadership as the most convenient way to maximize both availability and participation. The training sessions were conducted in situ within our ED.

Primary goals of the training sessions were to have participants practice their roles associated with the new process while working as a team, to recognize some difficulties associated with PPE that may not be routinely used, and to recognize the effectiveness of checklist and standardized processes. The secondary goal of the training sessions was to identify process changes that could be implemented successfully, as well as those requiring revisions or removal from the redesigned intubation process. Participants were allowed to practice as many times as desired, using actual equipment and an intubating mannequin.

Following each training session, a debriefing was held to help to ensure participant understanding of the material as well as to solicit their professional input into the redesigned system. Based on the observations and feedback of participants comprising the core team, numerous changes were made over a short period of time to enhance the algorithm. By the fourth day of training and study, there were no major changes identified for the algorithm and it was then trialed in our department.

Upon completion of the training session, participants were invited to complete an online (SurveyMonkey), seven-question survey focusing on reaction (Appendix A). The course evaluation was approved by our institution’s institutional review board (approval #PRO13040395). The link to the survey was emailed to participants. The survey consisted of basic information including role (physician, RT, RN), and prior use of PAPR for intubation, followed by four 5-point Likert-scale items (scored from strongly disagree to strongly agree with a neutral option) to assess the educational objectives of the session. Finally, there was one final, open-text item asking for any additional feedback or suggestions. To increase response rates, we sent another email five days later as a reminder to participants.

## RESULTS

Two intubations of real patients were carried out using the new system by two of us on the core team. (PP and GS). A post-procedural multidisciplinary debriefing was held and resulted in several more changes to the algorithm. The algorithm underwent a total of 17 iterations of substantial change. Following the live patient validation and subsequent adjustments, an online video training overview was created and bundled with a package of PDFs to create print materials. This allowed for dissemination across our health system to facilitate rapid implementation at facilities that had a perceived need for such a systematic change.

A total of 54 participants completed the training course over the initial seven sessions. We received 48 total responses (19 DRs, 28 RNs, and one RT), for a response rate of 88.8%. Survey results were largely positive. Specifically, there was a positive improvement in level of confidence with one’s role in the intubation process. Prior to the course, only 32.33% selected either “agree” or “strongly agree” to the item, “Prior to taking this course, I felt confident with my role in the intubation process of high-risk COVID patients.” However, after completing the course, 95.74% selected either “agree” or “strongly agree” to the item, “After completing this course, I feel confident with my role in the intubation process of high-risk COVID patients.” Further, 97.92% selected either “agree” or “strongly agree” to the item “I would recommend this course to other healthcare providers.” Most participants (93.75%) also felt that the course enhanced their team communication skills (question 6).

We received 18 responses for the open-ended item, with feedback overall positive. Many of the responses highlighted the usefulness of the training overall, expressing gratitude for the dedicated time to physically practice. One major theme, however, was the effectiveness of the multidisciplinary, team-based approach, which was highlighted by the following comments:

*Very educational. There was a lot of open discussion and suggestions were bounced back and forth which was nice*.

The Intubation Simulation was excellent! It was very helpful to have staff with different areas of expertise providing input from their experiences & suggesting ways to improve our performance & decrease our risk for an exposure. Thank you for taking the time to facilitate this!

*This was high yield, manageable length, and extremely team based. I’m glad we were able to do it within the clinical setting in which we work*.

Areas of improvement suggested from the open-ended feedback included using different scenarios to allow for more practice and providing a finalized list of the algorithm for those who participated early in the course before the final changes were implemented, the latter of which was satisfied with the online materials distributed across the health system.

## DISCUSSION

What is unique to our algorithm and training sessions is that we combined training with evaluation and iterative practice improvement into a rapid cycle re-design of traditional airway practices. We incorporated multidisciplinary practice and were able to incorporate the suggestions of practicing professionals near-real time for system optimization. Through the first four sessions, we made multiple revisions to the algorithm.

Examples of major revisions included methods for covering the tube after the intubation while the ventilator circuit was being attached. A number of other revisions were also made, addressing specific placement of equipment in the room and adding equipment to the “inside items” (marker for labeling RSI medications), as suggested by nurses. Finally, different methods of communication between the team inside the room and the nurse and PCT outside the room were also tested, with the final decision to use readily available baby monitors. The original solution of using Spectralink phones was found to be unsuccessful during the first of the trials involving an actual patient.

Although we did want to standardize the intubation process for patients with suspicion for COVID-19 as much as possible, the algorithm still allows some room for incorporating individual physician clinical decision-making. Recognizing different physician preferences in medications for RSI as well as post-intubation sedation, for example, we did not mandate the exact drug regimen in our algorithm. Selection of ETT size, method of backup plan, and placement of central or arterial line access were similarly addressed. Due to different approaches to management of a patient’s respiratory status, especially within the setting of rapidly evolving understanding of COVID-19 and its optimal management, we did not feel as though any criteria for intubation would follow a “one size fits all” mentality and should instead be considered on a case-by-case basis. Therefore, our algorithm begins after the need for intubation has been established. Our focus was on optimization of the process once the clinical decision to intubate was made.

We recognize that there have been many other proposed methods for minimizing the aerosolization of viral particles during intubation, in addition to other preferences for intubation techniques and use of backup devices. Some institutions have even incorporated specific intubation teams that intubate all high-risk patients in the hospital. We recognize that these are all acceptable strategies for addressing the common problem. We believe that the adoption of any one system is based upon the resources, experiences, and situations that are unique to the individual ED.

While we designed the details of our algorithm based on the resources available at our institution and what we determined to be the most feasible through feedback received during our training sessions, we tried to identify and include flexibility in areas we thought would have the most implementation variability. Therefore, our revised airway process recommendations could be easily adapted for use at other EDs. The basic structure and overall process are easily transferable, while specific materials and details could be adapted based on availability and preferences at other institutions. Implementation of our streamlined process could have profound effects on efficiency of patient care, patient safety, and safety of the healthcare team.

Lastly, our extensive process validation that included simulation training sessions along with debriefings after our first two experiences with actual patients allowed for the development of materials to facilitate deployment of our recommended new approach to airway management to the 19 EDs across our health system that see over 400,000 patients per year. In addition to our asynchronous training and print materials, we will be conducting train-the-trainer sessions in collaboration with WISER to help local champions adopt our methods for their institution efficiently and effectively.

## LIMITATIONS

One limitation of our work is the inability to evaluate our algorithm in a large number of actual patients. The formidable challenges imposed upon the delivery of healthcare during the pandemic combined with the need to maximize the safety of healthcare providers necessitated a rapid roll-out of our revised processes based on our findings from our rapid-cycle improvement methodology. However, we do intend to collect further feedback from real-time use. Another limitation to our report is that the analysis of our training sessions is limited to Kirkpatrick Level 1 reaction data. Given the demands of our team during the pandemic, combined with the changing details of our training sessions based on the iterative feedback, a more formal assessment was not feasible.

Future studies could address a more formal effectiveness of the training program, focusing on team-based, non-technical skills acquisition. A formal review of patient outcomes associated with the new airway management recommendations after implementation in the ED would also be appropriate.

## CONCLUSION

A multidisciplinary, team-based approach to the development and training of a standardized intubation algorithm combining simulation and a rapid-cycle improvement methodology is a useful, effective process to respond to rapidly evolving clinical information and experiences during a global pandemic.

## Supplementary Information
