# Supplementary material for: A Multidisciplinary Intubation Algorithm for Suspected COVID-19 Patients in the Emergency Department
Source: West J Emerg Med. 2020 Jun 3;21(4):764–70. doi: 10.5811/westjem.2020.5.47835 (PMC7390585; doi:10.5811/westjem.2020.5.47835)
Supplement: Supplementary file 1 [file wjem-21-764-s001.docx]

**Appendix A**

1. What is your role in the ED?

____Resident Physician        ____ RN       ____ Other (please specify)

____Attending Physician       ____ RT

2. Prior to taking this course, how many times have you used a PAPR?

0                              1-2                              3-5                             6-9                               >10

Please use the following scale for the questions below:

     1                           2                             3                               4                              5

Strongly               Disagree                 Neutral/                      Agree                       Strongly

Disagree                                             No Opinion                                                  Agree

| 3.Prior to taking this course, I felt confident with my role in the intubation process of high-risk COVID patients. | 1 | 2 | 3 | 4 | 5 |
| --- | --- | --- | --- | --- | --- |
| 4.After completing this course, I feel confident with my role in the intubation process of high-risk COVID patients. | 1 | 2 | 3 | 4 | 5 |
| 5.I would recommend this course to other healthcare providers. | 1 | 2 | 3 | 4 | 5 |
| 6. This course enhanced my team communication skills. | 1 | 2 | 3 | 4 | 5 |

Please provide any additional comments or suggestions for improvement.

____________________________________________________________________________________________________________________________________________________

____________________________________________________________________________________________________________________________________________________

__________________________________________________________________________
